# Supplementary material for: QTG-Miner aids rapid dissection of the genetic base of tassel branch number in maize
Source: Nat Commun. 2023 Aug 26;14:5232. doi: 10.1038/s41467-023-41022-1 (PMC10460418; doi:10.1038/s41467-023-41022-1)
Supplement: Supplementary file 1 — Supplementary Information [file 41467_2023_41022_MOESM1_ESM.pdf]

QTG-Miner aids rapid dissection of the genetic base of tassel branch number in maize

Wang *et al.*

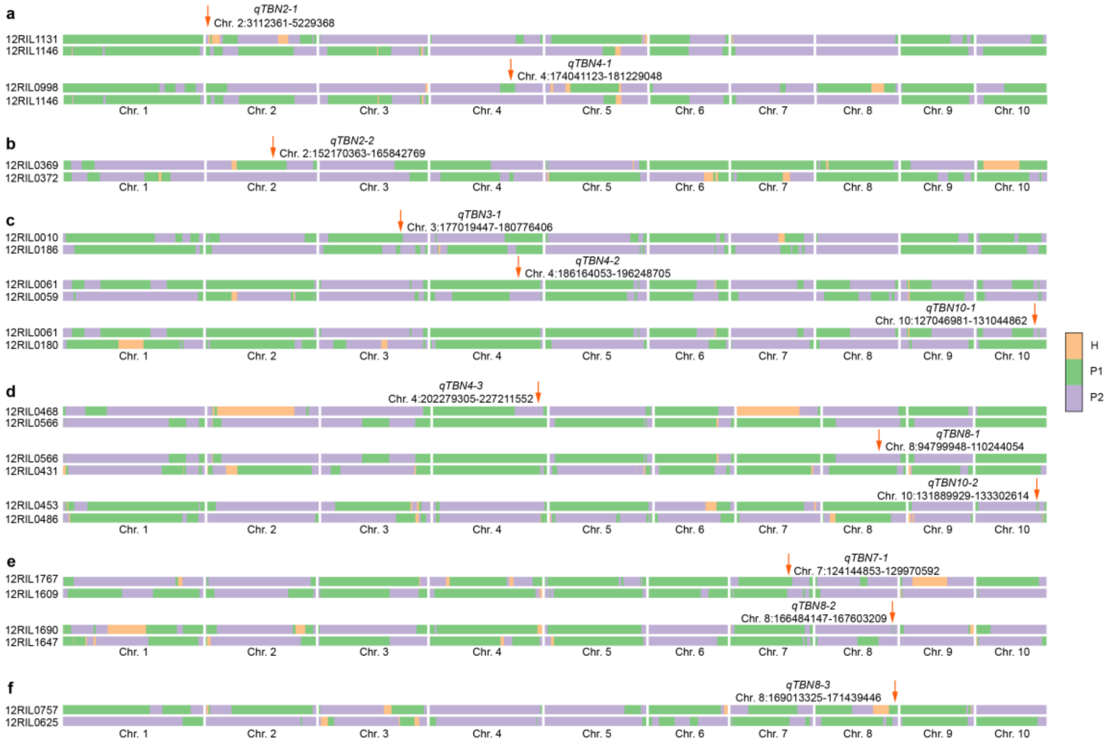

Supplementary Figure 1. Genotype information of 12 single QTL-paired RILs. 12 TBN QTLs derived from 6 recombination inbred line (RILs) populations. Detailed genotype information of 12 single QTL-paired RILs were showed in **a**-**f**. **a,** Genotype of RILs for *qTBN2-1* and *qTBN4-1* identified from ZHENG58/SK populations. **b,** Genotype of RILs for *qTBN2-2* identified from KUI3/B77 populations. **c,** Genotype of RILs for *qTBN3-1*, *qTBN4-2* and *qTBN10-1* identified from B73/BY804 populations. **d,** Genotype of RILs for *qTBN4-3*, *qTBN8-1* and *qTBN10-2* identified from DAN340/K22 populations. **e,** Genotype of RILs for *qTBN7-1* and *qTBN8-2* identified from DE3/BY815 populations. **f,** Genotype of RILs for *qTBN8-3* identified from K22/CI7 populations.

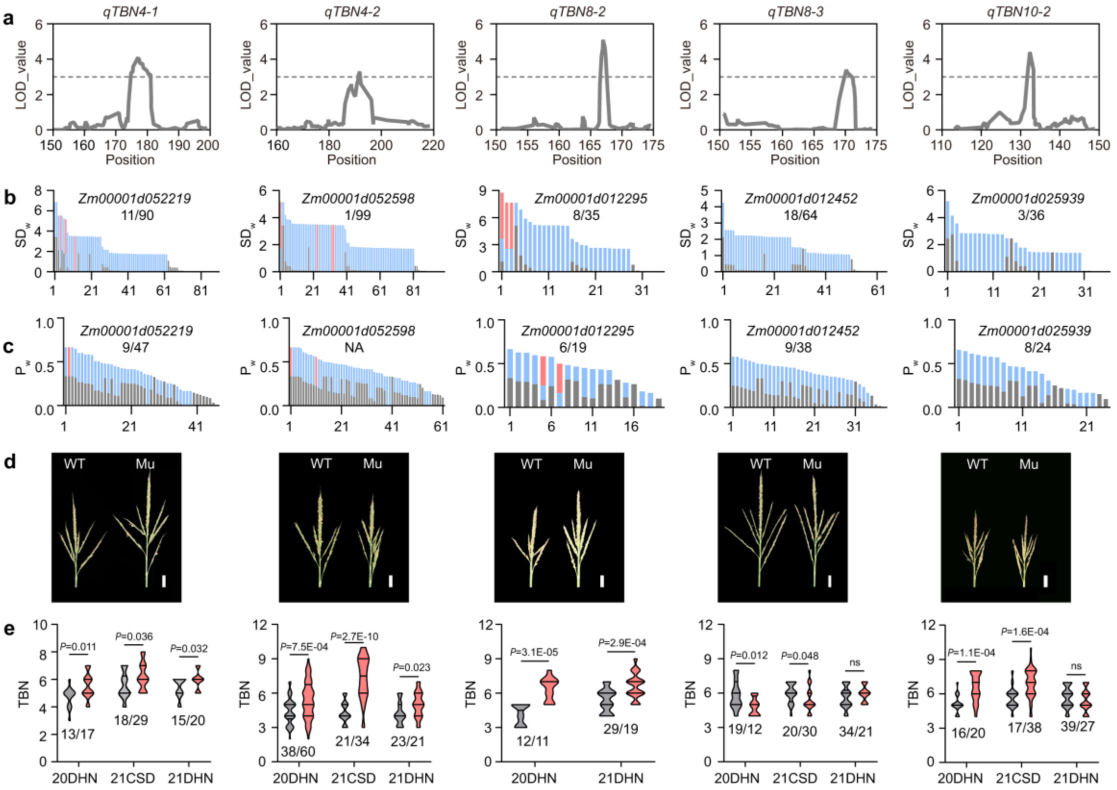

Supplementary Figure 2. Verification of candidate genes identified by QTG-Miner for other five TBN QTLs in maize. a, Primary genetic mapping results of other five TBN QTLs. **b,** Detailed weighted SD values (SD_w) of genes in the five TBN QTL intervals. Pink indicates SD_d, light blue indicates SD_v, and gray indicates SD_g. **c,** Detailed weighted_Probability (P_w) of genes in the five TBN QTL intervals. Pink indicates P_d, light blue indicates P_v, and gray indicates P_g. **d,** Representative photographs showing the TBN phenotype in wild type (left) and mutant (right). Scale bars, 2 cm. **e,** TBN in wild type (gray violin plots) and mutants (pink violin plots). 20DHN, Hainan in winter 2020. 21CSD, Shandong in spring 2021. 21DHN, Hainan in winter 2021. Violin plots: median ± upper and lower quartiles. P values were determined by two-sided Student's t-tests. $*P < 0.05$, $**P < 0.01$, $***P < 0.001$. Source data of Supplementary Fig. 2e are provided as a Source Data file.

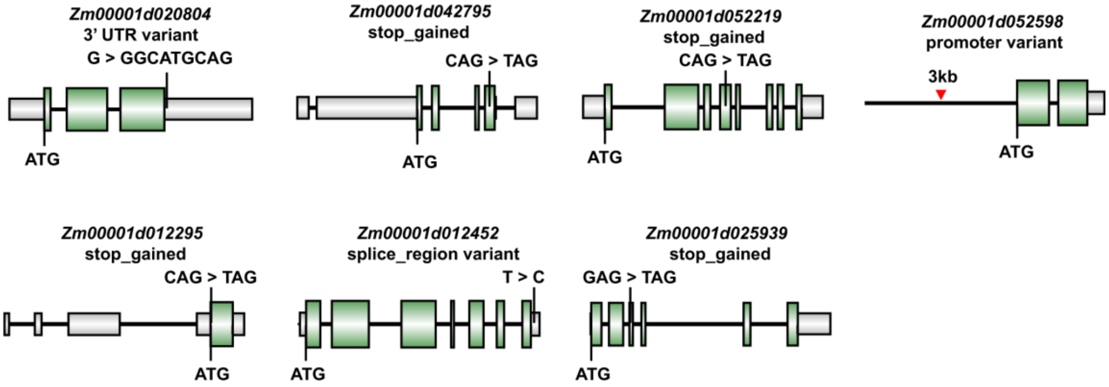

Supplementary Figure 3. DNA sequence comparison of seven verified genes between parental lines.

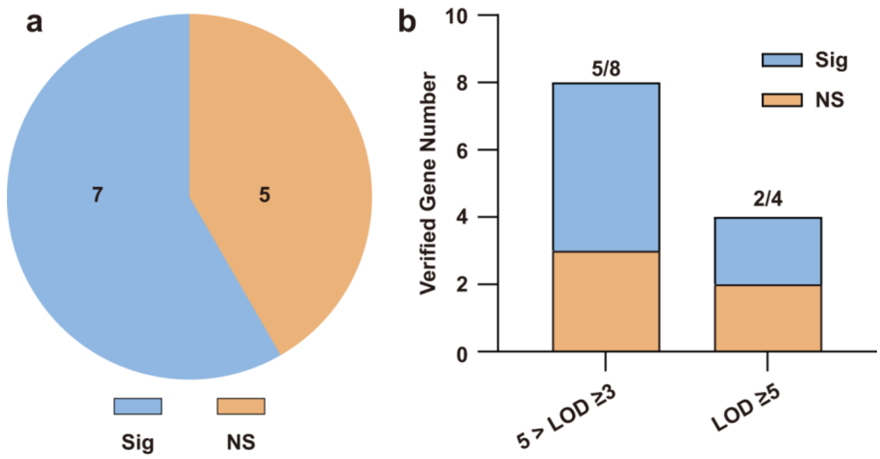

Supplementary Figure 4. Success rate of fine mapping of TBN QTLs by QTG-Miner. **a,** Overall proportion of successfully validated genes (Sig) and not validated genes (NS) prioritized by QTG-Miner. **b,** Validation rate of TBN QTLs with different LOD values.

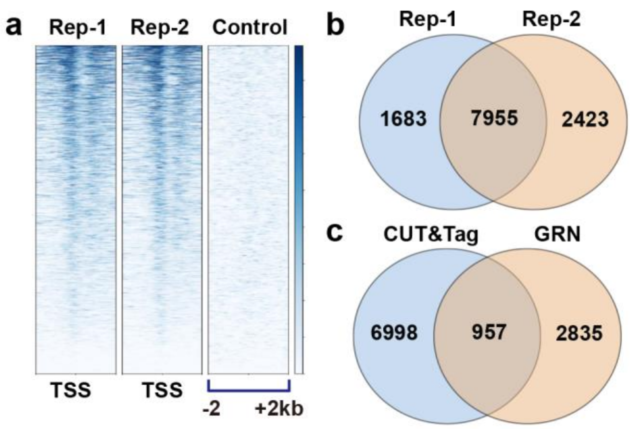

Supplementary Figure 5. Identification of ZmHD-ZIP120 target genes. a, Heatmap representation of tsCUT&Tag signals ~2,000 bp upstream and downstream of the transcription start sites (TSS) of ZmHD-ZIP120 target genes. Rep, biological replicate. Control, empty vector of GFP (Green Fluorescent Protein). **b,** Shared target genes between Rep-1 and Rep-2 for ZmHD-ZIP120 targets. Rep, biological replicate. **c,** Overlapping target genes defined by tsCUT&Tag and the GRN for ZmHD-ZIP120.

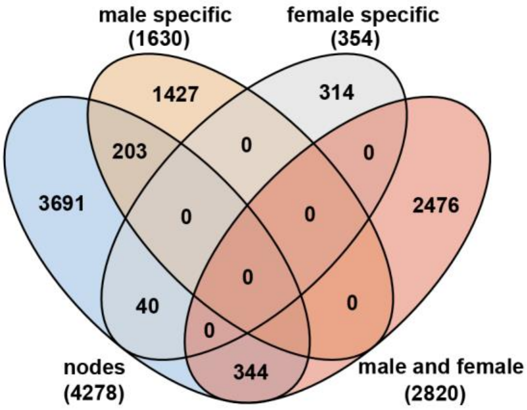

Supplementary Figure 6. Venn diagram showing the extent of overlap among node genes from the TBN network, male-specific selected genes, female-specific selected genes and co-selected genes between MHGs and FHGs.

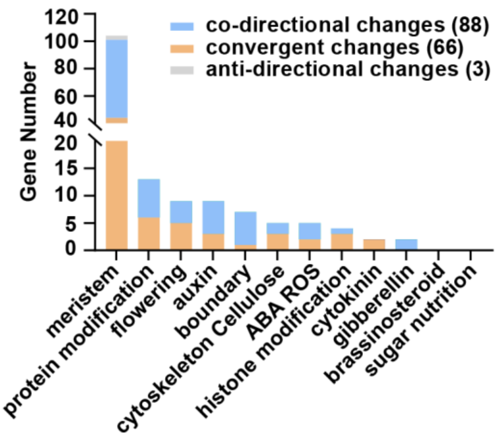

Supplementary Figure 7. Distribution of three groups of selected genes across 12 biological pathways.

Supplementary Table 1. AUC values of the results for five ML algorithms.

Replicates	NeuralNet	LR	XgBoost	Bagging	SVM
1	0.9753	0.9383	0.8889	0.9198	0.4691
2	0.9740	0.9351	0.9481	0.5584	0.3636
3	0.8625	0.8500	0.8750	0.8750	0.6375
4	0.9464	0.9464	0.9286	0.9286	0.8036
5	0.8831	0.8701	0.8571	0.8701	0.7662
6	0.9740	0.9351	0.9221	0.9416	0.8182
7	0.9464	0.9107	0.8036	0.8125	0.6964
8	0.9306	0.8750	0.7778	0.7986	0.7917
9	0.9000	0.8000	0.8750	0.7750	0.7875
10	0.8889	0.9444	0.8750	0.8611	0.7361
11	0.9481	0.8442	0.9221	0.8766	0.8831
12	0.9722	0.9167	0.9722	0.9722	0.7778
13	0.9091	0.9091	0.9545	0.7792	0.9091
14	0.9821	1.0000	1.0000	0.9375	0.3036
15	0.8889	0.8194	0.7222	0.7917	0.9167
16	0.8571	0.8571	0.8571	0.8929	0.6429
17	0.8750	0.8375	0.7875	0.5813	0.8000
18	0.9861	1.0000	0.9861	0.9444	0.9028
19	0.8889	0.8333	0.7917	0.7431	0.7500
20	0.9375	0.9375	0.8750	0.8313	0.7375

Supplementary Table 2. Primers used in this study.

Purpose	Primer name	Primer (5'-3')
EMS	1988EMS-F	AACCACTTTCCTAACTTGATG
EMS	1988EMS-R	AACAATAAACCATCCCAGTAA
EMS	5315EMS-F	CACCACAGAATCAAAGAAAAGC
EMS	5315EMS-R	GTAGGGGTTGGGGTCGTAGT
EMS	2795EMS-F	ACTTAGCAACAAGAACCCACA
EMS	2795EMS-R	ACAAAAGTTCTGTCTAAAAAATC
EMS	2219EMS-F	TCGCAACTCTCAAAGCCTACT
EMS	2219EMS-R	ACGGGTGTTTATCGCATTCTT
EMS	2598EMS-F	CTTAGAACGCATATACGACAC
EMS	2598EMS-R	TGCGTACGGGTGGTGGTGA
EMS	3059EMS-F	GTTTATCCACTTGTGACCTGA
EMS	3059EMS-R	TGAAAAACAAACTTGGTCTCT
EMS	0271EMS-F	TGTCCGTCTCTACGCTAAAGG
EMS	0271EMS-R	CAGGGGAGTTCTCTACAGCAC
EMS	2295EMS-F	TGGCGTGAAAAAACTTGTGCT
EMS	2295EMS-R	GCAAAGAAAAAGAGAAAGAGGC
EMS	2452EMS-F	CGTAACAGCATCAGTATCAAAT
EMS	2452EMS-R	GGACTCAAGAATGACGGGC
EMS	5750EMS-F	ATCTTCAAGAAAATAAAACATGCTG
EMS	5750EMS-R	GTTGGTAACGAGTGCTCCCTA
EMS	5939EMS-F	TTCGGAAACAAAACATCTGG
EMS	5939EMS-R	GGTATGCCTGCTCACAGATTC
CRISPR	2795CR-F	CACTGCTGCTTATCTCGTGGC
CRISPR	2795CR-R	CAGTGGTTGGTGGCTACGCTA
CRISPR	0804CR-F	CGGACGAGGAGATGATGGC
CRISPR	0804CR-R	CCTCTCGCTCCGAATTCTTTAT
qPCR	2795-qF	TGTGCTCGTTGGTGAGACTGC
qPCR	2795-qR	TTCAATTTTAGCTTCCTTGGAGT
qPCR	0804-qF	GAGCTTCACGTCGGTCAAGG
qPCR	0804-qR	CGCGTCAAGAAGAGTACCCC
qPCR	Actin-qF	GCCGAGCGAGAAATTGTAAG
qPCR	Actin-qR	TGGTGATTACTTGGCCATCA
