# Supplementary material for: QTG-Miner aids rapid dissection of the genetic base of tassel branch number in maize
Source: Nat Commun. 2023 Aug 26;14:5232. doi: 10.1038/s41467-023-41022-1 (PMC10460418; doi:10.1038/s41467-023-41022-1)
Supplement: Supplementary file 3 — Description of Additional Supplementary Files [file 41467_2023_41022_MOESM3_ESM.pdf]

Description of Additional Supplementary Files

File Name: Supplementary Data 1
Description: Information on the 12 TBN QTLs analyzed in this study

File Name: Supplementary Data 2
Description: Genotype information of single QTL-paired RILs derived from inbred lines ZHENG58 and SK

File Name: Supplementary Data 3
Description: Genotype information of single QTL-paired RILs derived from inbred lines KUI3 and B77

File Name: Supplementary Data 4
Description: Genotype information of single QTL-paired RILs derived from inbred lines B73 and BY804

File Name: Supplementary Data 5
Description: Genotype information of single QTL-paired RILs derived from inbred lines DAN340 and K22

File Name: Supplementary Data 6
Description: Genotype information of single QTL-paired RILs derived from inbred lines BY815 and DE3

File Name: Supplementary Data 7
Description: Genotype information of single QTL-paired RILs derived from inbred lines K22 and CI7

File Name: Supplementary Data 8
Description: Summary of RNA-seq analysis.

File Name: Supplementary Data 9
Description: DEGs associated with 12 TBN QTLs

File Name: Supplementary Data 10
Description: Sequence variants associated with 12 TBN QTLs

File Name: Supplementary Data 11
Description: Positive and negative TBN genes

File Name: Supplementary Data 12
Description: List of EMS-mutagenized materials used for assessing candidates for the 12 TBN QTLs

File Name: Supplementary Data 13
Description: List of CRISPR-edited materials used for assessing candidates for the 12 TBN QTLs

File Name: Supplementary Data 14
Description: List of 72 genes used for network reconstruction of TBN traits

File Name: Supplementary Data 15
Description: DEGs identified from six EMS-mutagenized materials

File Name: Supplementary Data 16

Description: Potential target genes of ZmHD-ZIP120

File Name: Supplementary Data 17
Description: Enriched GO terms among 4,278 genes

File Name: Supplementary Data 18
Description: Enriched GO terms among co-selection genes

File Name: Supplementary Data 19
Description: Enriched GO terms among male group specific genes

File Name: Supplementary Data 20
Description: Enriched GO terms among co-directional genes

File Name: Supplementary Data 21
Description: Enriched GO terms among convergent genes
