# Supplementary material for: QTG-Miner aids rapid dissection of the genetic base of tassel branch number in maize
Source: Nat Commun. 2023 Aug 26;14:5232. doi: 10.1038/s41467-023-41022-1 (PMC10460418; doi:10.1038/s41467-023-41022-1)
Supplement: Supplementary file 25 — Reporting Summary [file 41467_2023_41022_MOESM25_ESM.pdf]

nature portfolio

Corresponding author(s): Lin Li

Last updated by author(s): 2023-08-14

Reporting Summary

Nature Portfolio wishes to improve the reproducibility of the work that we publish. This form provides structure for consistency and transparency in reporting. For further information on Nature Portfolio policies, see our Editorial Policies and the Editorial Policy Checklist.

Statistics

For all statistical analyses, confirm that the following items are present in the figure legend, table legend, main text, or Methods section.

n/a	Confirmed	
☐	☒	The exact sample size (n) for each experimental group/condition, given as a discrete number and unit of measurement
☐	☒	A statement on whether measurements were taken from distinct samples or whether the same sample was measured repeatedly
☐	☒	The statistical test(s) used AND whether they are one- or two-sided<br>*Only common tests should be described solely by name; describe more complex techniques in the Methods section.*
☐	☒	A description of all covariates tested
☒	☐	A description of any assumptions or corrections, such as tests of normality and adjustment for multiple comparisons
☐	☒	A full description of the statistical parameters including central tendency (e.g. means) or other basic estimates (e.g. regression coefficient) AND variation (e.g. standard deviation) or associated estimates of uncertainty (e.g. confidence intervals)
☐	☒	For null hypothesis testing, the test statistic (e.g. F, t, r) with confidence intervals, effect sizes, degrees of freedom and P value noted<br>*Give P values as exact values whenever suitable.*
☒	☐	For Bayesian analysis, information on the choice of priors and Markov chain Monte Carlo settings
☒	☐	For hierarchical and complex designs, identification of the appropriate level for tests and full reporting of outcomes
☒	☐	Estimates of effect sizes (e.g. Cohen's d, Pearson's r), indicating how they were calculated

Our web collection on statistics for biologists contains articles on many of the points above.

Software and code

Policy information about availability of computer code

Data collection	No special software was used
Data analysis	Machine learning algorithms are uploaded to Github (https://github.com/hanlinqian/IntegrativeNetworkMap/tree/INM/Section5)<br>Shortest distance algorithms are uploaded to Github (https://github.com/hanlinqian/TBNnetwork)<br>Softwares:<br>1.Hisat2, version 2.2.0<br>2. cuffdiff, version 2.2.1<br>3.GATK, version 3.7<br>4. SnpEff, version 4.3t<br>5.VCFtools , version 0.1.16<br>6.Gephi, version 0.9.2<br>7. agriGO, v.2.0<br>8.scikit-learn-o.21.2

For manuscripts utilizing custom algorithms or software that are central to the research but not yet described in published literature, software must be made available to editors and reviewers. We strongly encourage code deposition in a community repository (e.g. GitHub). See the Nature Portfolio guidelines for submitting code & software for further information.

All manuscripts must include a data availability statement. This statement should provide the following information, where applicable:
- Accession codes, unique identifiers, or web links for publicly available datasets
- A description of any restrictions on data availability
- For clinical datasets or third party data, please ensure that the statement adheres to our policy

Data

Policy information about availability of data

Sequence data discussed in this article can be found in the MaizeGDB (https://www.maizegdb.org/) gene records under the following accession numbers: qTBN3-1 (Zm00001d042795), qTBN4-1 (Zm00001d052219), qTBN4-2 (Zm00001d052598), qTBN7-1 (Zm00001d020804), qTBN8-2 (Zm00001d012295), qTBN8-3 (Zm00001d012452), qTBN10-2 (Zm00001d025939). Maize reference genome(B73 RefGen_v4.36) was downloaded from the link (ftp://ftp.ensemblgenomes.org/pub/release-36/plants/fasta/zea_mays/dna/Zea_mays.AGPv4.dna.toplevel.fa.gz).
The raw sequencing data generated in this paper have been deposited in the Genome Sequence Archive76 in National Genomics Data Center77, China National Center for Bioinformation / Beijing Institute of Genomics, Chinese Academy of Sciences (GSA: CRA009203) that are publicly accessible at https://ngdc.cncb.ac.cn/gsa.
Source data are provided with this paper.

Research involving human participants, their data, or biological material

Policy information about studies with human participants or human data. See also policy information about sex, gender (identity/presentation), and sexual orientation and race, ethnicity and racism.

Reporting on sex and gender	not applicable
Reporting on race, ethnicity, or other socially relevant groupings	not applicable
Population characteristics	not applicable
Recruitment	not applicable
Ethics oversight	not applicable

Note that full information on the approval of the study protocol must also be provided in the manuscript.

Field-specific reporting

Please select the one below that is the best fit for your research. If you are not sure, read the appropriate sections before making your selection.

☒ Life sciences ☐ Behavioural & social sciences ☐ Ecological, evolutionary & environmental sciences

For a reference copy of the document with all sections, see nature.com/documents/nr-reporting-summary-flat.pdf

Life sciences study design

All studies must disclose on these points even when the disclosure is negative.

Sample size	The sample size was determined according to the reports in the related research subjects. For the phenotype statistic for the EMS-mutagenized and CRISPR-edited materials, at least 10 samples in full maturity were investigated in field for the students' t test analysis in determining the phenotype traits. For RNA-seq experiment, immature tassel tissues 2–4 mm in length from at least six independent plants were collected, and two independent replicates per genotype. No statistical methods were used to predetermine sample sizes.
Data exclusions	No data were excluded from the analyses.
Replication	For each phenotype investigation experiment, at least two independently biological experiments were repeated.
Randomization	Samples were allocated into different experimental groups according to the genotypes.
Blinding	The investigators were blinded to group allocation during data collection and analysis.

Reporting for specific materials, systems and methods

We require information from authors about some types of materials, experimental systems and methods used in many studies. Here, indicate whether each material, system or method listed is relevant to your study. If you are not sure if a list item applies to your research, read the appropriate section before selecting a response.

Materials & experimental systems

n/a	Involved in the study	
☒	☐	Antibodies
☒	☐	Eukaryotic cell lines
☒	☐	Palaeontology and archaeology
☒	☐	Animals and other organisms
☒	☐	Clinical data
☒	☐	Dual use research of concern
☐	☒	Plants

Methods

n/a	Involved in the study	
☒	☐	ChIP-seq
☒	☐	Flow cytometry
☒	☐	MRI-based neuroimaging

Dual use research of concern

Policy information about dual use research of concern

Hazards

Could the accidental, deliberate or reckless misuse of agents or technologies generated in the work, or the application of information presented in the manuscript, pose a threat to:

No	Yes	
☒	☐	Public health
☒	☐	National security
☒	☐	Crops and/or livestock
☒	☐	Ecosystems
☒	☐	Any other significant area

Experiments of concern

Does the work involve any of these experiments of concern:

No	Yes	
☒	☐	Demonstrate how to render a vaccine ineffective
☒	☐	Confer resistance to therapeutically useful antibiotics or antiviral agents
☒	☐	Enhance the virulence of a pathogen or render a nonpathogen virulent
☒	☐	Increase transmissibility of a pathogen
☒	☐	Alter the host range of a pathogen
☒	☐	Enable evasion of diagnostic/detection modalities
☒	☐	Enable the weaponization of a biological agent or toxin
☒	☐	Any other potentially harmful combination of experiments and agents
